# Tool to assess risk of bias in studies estimating the prevalence of mental health disorders (RoB-PrevMH)

**DOI:** 10.1136/bmjment-2023-300694

**Published:** 2023-10-29

**Authors:** Thomy Tonia, Diana Buitrago-Garcia, Natalie Luise Peter, Cristina Mesa-Vieira, Tianjing Li, Toshi A Furukawa, Andrea Cipriani, Stefan Leucht, Nicola Low, Georgia Salanti

**Affiliations:** 1 Institute of Social and Preventive Medicine, University of Bern, Bern, Switzerland; 2 Graduate School of Health Sciences, University of Bern, Bern, Switzerland; 3 Department of Psychiatry and Psychotherapy, Klinikum rechts der Isar, School of Medicine and Health, Technical University of Munich, München, Germany; 4 Department of Ophthalmology, School of Medicine, University of Colorado Anschutz Medical Campus, Aurora, Colorado, USA; 5 Department of Health Promotion and Human Behavior, Kyoto University Graduate School of Medicine / School of Public Health, Kyoto, Japan; 6 Department of Psychiatry, University of Oxford, Oxford, UK; 7 Oxford Precision Psychiatry Lab, NIHR Oxford Health Biomedical Research Centre, Oxford, UK; 8 Oxford Health NHS Foundation Trust, Warneford Hospital, Oxford, UK; 9 Department of Psychiatry and Psychotherapy, Klinikum rechts der Isar, School of Medicine, Technical University of Munich, Freising, Germany

## Abstract

**Objective:**

There is no standard tool for assessing risk of bias (RoB) in prevalence studies. For the purposes of a living systematic review during the COVID-19 pandemic, we developed a tool to evaluate RoB in studies measuring the prevalence of mental health disorders (RoB-PrevMH) and tested inter-rater reliability.

**Methods:**

We decided on items and signalling questions to include in RoB-PrevMH through iterative discussions. We tested the reliability of assessments by different users with two sets of prevalence studies. The first set included a random sample of 50 studies from our living systematic review. The second set included 33 studies from a systematic review of the prevalence of post-traumatic stress disorders, major depression and generalised anxiety disorder. We assessed the inter-rater agreement by calculating the proportion of agreement and Kappa statistic for each item.

**Results:**

RoB-PrevMH consists of three items that address selection bias and information bias. Introductory and signalling questions guide the application of the tool to the review question. The inter-rater agreement for the three items was 83%, 90% and 93%. The weighted kappa scores were 0.63 (95% CI 0.54 to 0.73), 0.71 (95% CI 0.67 to 0.85) and 0.32 (95% CI −0.04 to 0.63), respectively.

**Conclusions:**

RoB-PrevMH is a brief, user-friendly and adaptable tool for assessing RoB in studies on prevalence of mental health disorders. Initial results for inter-rater agreement were fair to substantial. The tool’s validity, reliability and applicability should be assessed in future projects.

## Background

Studies of prevalence provide essential information for estimating the burden of mental health conditions, which can inform research and policy-making.^1^ The pandemic of COVID-19, a disease first described in 2020,^2^ rapidly generated a large volume of literature,^3^ about studies on the prevalence of a wide range of conditions, including those related to mental health. Increased levels of anxiety, depression, psychological distress, as well as an increase in violent behaviour, alcohol and substance use, among others have been described in association with fear of infection and the effects of contamination measures.^1 4^ Temporary relief from obligations at school or work, or the need to commute, on the other hand, might alleviate stress for some populations.^1^

A systematic review provides a structured way to gather, assess and synthesise evidence from prevalence studies. One essential step in performing a systematic review is the assessment of risk of bias (RoB) of the included studies^5^ because the potential biases affect how certain we are about the included evidence and its interpretation.^6^ There is no agreement on how to assess RoB in prevalence studies,^7^ despite a 10-fold increase in systematic reviews of prevalence studies in the last decade.^7 8^ Substantial variability exists in how RoB in prevalence studies have been assessed with more than 30 tools identified and several judged to be inappropriate.^9^ Notably, some questions/items in existing tools focus on the quality of reporting which makes not possible to assess the biases present in prevalence studies.

To overcome the shortcomings of previous tools, such as distinguishing between RoB and quality of reporting and being adaptable to different questions, the purpose of this paper is to present a RoB tool developed to evaluate RoB in studies measuring the prevalence of mental health disorders (RoB-PrevMH). We describe the steps for developing this tool, its items, and the results of inter-rater agreement obtained by applying the tool to two sets of prevalence studies on mental health disorders.

## Methods

RoB-PrevMH was developed within the MHCOVID project (https://mhcovid.ispm.unibe.ch/), a living systematic review assessing the effect of the COVID-19 pandemic and the containment measures on mental health of the population.^1 4 10^ MHCOVID involves many volunteers recruited through crowdsourcing to help with data extraction and RoB assessment of a large volume of literature (referred to as the MHCOVID Crowd). We prioritised brevity and ease of application in developing the tool, owning to the different backgrounds and levels of experience and expertise of MHCOVID Crowd members in the assessment of RoB.

### Development of the tool

We searched Medline and Embase (Ovid) from inception to September 2020 to identify published tools or checklists designed to assess the quality, RoB, and quality of reporting in prevalence studies (online supplemental appendix 1). In addition, we searched the Equator network website (https://www.equator-network.org/) and a database of systematic reviews of prevalence studies.^11^ One researcher (DBG) screened the search results to identify relevant tools that assessed RoB in prevalence studies.

10.1136/bmjment-2023-300694.supp1 Supplementary data

We extracted the items from each tool selected for inclusion and grouped them under the domains of selection bias and information bias. For selection bias, items from the existing tools were separated into those referring to population representativeness or to ‘the proportion of respondents’. For information bias, items from the existing tools were separated into those referring to observer bias, recall bias or misclassification bias. Items not related to the named biases were tagged as ‘other bias’ or ‘reporting’.

**Figure 1:**
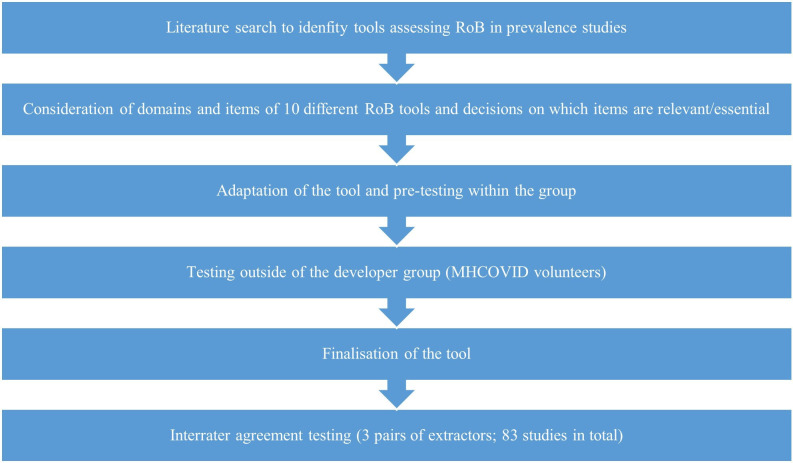
Process of developing and testing RoB-PrevMH.

Five researchers (DBG, NL, NLP, GS and TT) individually went through the list of questions in each included tool, excluded duplicated questions, and marked those that were most relevant for prevalence studies for mental health disorders. They then discussed their assessments and reached consensus prior to drafting the first version of the tool and the signalling questions. Figure 1 illustrates the process of developing RoB-PrevMH.

### Testing and finalisation of the tool

Four members of the team (SL, NLP, GS and TT) pilot tested the first version of the tool and drafted a guidance document. Subsequently, these four researchers and four volunteers from the MHCOVID Crowd (who were not involved in the development of the tool) further tested the tool in a total of eight studies. Based on feedback from this exercise, the guidance document was updated accordingly, including examples and practical advice.

### Inter-rater reliability

We tested the reliability of assessments by different users of RoB-PrevMH with two sets of prevalence studies. The first set included 50 prevalence studies (two sets of 25) randomly selected from those identified as potentially relevant for the MHCOVID project during the abstract screening stage. Two pairs of researchers independently applied RoB-PrevMH (team A, 25 studies: CMV and TT; team B 25 studies: DBG and NLP). The second set included 33 studies from a systematic review of the prevalence of post-traumatic stress disorders, major depressive disorder and generalised anxiety disorder in migrants with premigration exposure to armed conflict.^12^ By using this second set of studies, we examined how RoB-PrevMH performed in a research question that was different from the one it was originally developed for. Two researchers (team C: DBG and CMV) independently applied RoB-PrevMH in this set of studies.

To assess reproducibility, we calculated the unweighted and weighted kappa statistic (with 95% CI). For weighted kappa, the observed and expected proportions of agreement are modified to measure the agreement among the ordered levels of bias (low, unclear, high) by assigning a weight of 0 to complete disagreement (rating low vs high RoB), 1 to perfect agreement and 0.5 for partial disagreement (ratings low vs unclear or high vs unclear).^13 14^ We also calculated the percentage of agreement between raters (number of agreements/number of assessments x 100). The analysis was conducted in STATA V.15.1^15^ . We followed the interpretation of the kappa statistic proposed by Landis and Koch (1977) and described in the STATA manual where the values below the cut points 0.00, 0.20, 0.40, 0.60, 0.80 and 1.00 approximately define poor, slight, fair, moderate substantial and almost perfect agreement.^16^

## Results

### Description of RoB-PrevMH tool

We identified 10 tools that assess RoB in prevalence studies, summarised in table 1.^13–22^ Following the process mentioned above, we developed RoB-PrevMH which consists of one introductory question and three items (table 2). It also includes signalling questions aimed to help the user reach a judgement; after completing our study we improved and refined the questions associated with two items and these are presented in table 2 alongside the original questions. The elaboration and guidance document is presented in online supplemental appendix 2. RoB for each item can be judged as ‘high’, ‘low’ or ‘unclear’. We instructed users to avoid judging any of the questions as unclear, whenever possible. This recommendation was based on the guidelines to assess the risk bias for Systematic Reviews on Interventions, which states that ‘unclear’ should be only used when the information about the domain is truly unknown.^23^ The tool does not allow a summary RoB assessment because some aspects of study quality might be more important than others, making aggregated scores problematic.^24 25^

10.1136/bmjment-2023-300694.supp2 Supplementary data

**Table 1:** RoB tools considered for developing RoB-PrevMH

ID	Tool	Description	No of items/questions	Validation process
1	Leboeuf-Yde and Lauritsen^17^ 1995	A tool designed to assess the quality of prevalence studies on low back pain.	Eleven methodological criteria	Not reported
2	Loney *et al* 18 1998	A critical appraisal tool designed to assess the methodological strengths, results and relevance of articles on prevalence of a health problem.	Eight items with a scoring system	Consensus between two assessors
3	Boyle^19^ 1998	A guideline to critically appraise prevalence studies on psychiatric disorders, both in the general population and in specific clinical settings.	Evaluates three main items divided in 11 questions	Not reported
4	Silva *et al* 20 2001	A tool to assess the usefulness of prevalence studies in the context of surveillance activities.	Covers six technical aspects divided in 19 questions with a scoring system	Consensus for the scoring system
5	Shamliyan *et al* 13 2010	A tool for evaluating the quality of studies that examine the prevalence of chronic conditions or risk factors.	Six criteria for external validity and five for internal validity	The tool was tested in four studies of incidence or prevalence. Kappa values showed fair agreement.
6	Hoy *et al* 14 2012	A risk of bias tool for prevalence studies based on Leboeuf-Yde and Lauritsen^17^ 1995.	Ten items plus a summary assessment.	Overall inter-rater agreement=91% Kappa=0.82 (95% CI 0.76 to 0.86)
7	Giannakopoulos *et al* 15 2012	An instrument for the qualitative assessment of the methodology of prevalence studies.	Ten items with a scoring system	Pilot phase Kappa for the quality score=mean 0.62±0.15 Kappa for individual questions=mean 0.78±0.27 After feedback Kappa for the quality score=range 0.94–1.00
8	Munn *et al* 16 2014	A critical appraisal tool for assessing studies included in systematic reviews of prevalence.	Ten questions	5-point Likert scale (one very unacceptable, 5 very acceptable) Ease of tool use=mean 3.63±0.72 Acceptability=mean 4.33±0.49 Timeliness=mean 3.94±0.57
9	The Joanna Briggs Institute^21^ 2016	A tool to assess the methodological quality of a prevalence study and the possibility of bias.	Nine questions with an overall appraisal question.	Not reported
10	Pega *et al* 22 2019	A tool for assessing the risk of bias in prevalence studies of exposure to occupational risk factors.	Eight domains	Using a raw measure of agreement, the tool achieved substantial agreement in six domains (conflict of interest, other bias, lack of blinding of study personnel, exposure misclassification, selective reporting of exposures) and poor agreement in two domains (incomplete exposure data, selection of participants into the study).

**Table 2:** Items included in RoB-PrevMH, suggested rephrasing and guidance

Item	Question	Elaboration
Domain: selection bias		
1. Representativeness of the sampling frame	Was the sample invited to participate in the study a true or close representation of the target population?	This question is about how well the people invited to participate in the study match the target population in demographic or clinical characteristics that are believed to be associated with the measured condition.
2. Representativeness of the responders	How would you rate the risk of non-response bias?	This question is about the characteristics and assumed presence of the condition in people who were invited but did not respond in a way that enabled the investigators to measure the condition of interest (not necessarily providing complete data).
	Suggested rephrasing: Was the sample that provided data a true or close representation the sample invited to participate?	
Domain: information bias		
3. Measurement of the condition	How do you judge the risk of information bias?	This question is about the appropriateness and reliability of the instrument or method used to measure the condition among people who provided the relevant data. Bias might occur when the training of observers of the outcome was not done or the procedures to collect the data are not the same for every participant or every timepoint included in the study. Bias might also occur when questions refer to the past and their answering depends on the condition.
	Suggested rephrasing: Was the condition measured/detected in an unbiased and reproducible way for all participants? Additional specific questions depending on the context: Was the tool used to measure the condition validated? Were the methods for measuring the condition standardised? Does the measurement of the condition depend on the memory of the participants?	

Each of the three items can be given a judgement high, low or unclear.

*This was a requirement for inclusion in the MHCOVID study and hence not included in the current version of the tool.^4^

The introductory question is ‘Was the target population clearly defined?’ By ‘target population’ we refer to the entire population for which we are interested to draw inference. In the first set of studies from the MHCOVID project, the target population of the systematic review was defined as ‘the general population’ or any age or gender-based subgroups of the general population (eg, children only, or men only, or elderly, see online supplemental appendix 2). In the second set of studies, the target population of the systematic review was migrants exposed to armed conflict.^25^

This introductory question had two response options; ‘yes’ or ‘no’ and has implications for the evaluation of the first RoB item: if the answer is ‘no’, the first item of the tool is automatically assigned an ‘unclear’ risk.

#### Item 1 selection bias: representativeness of the sampling frame

The first RoB item is related to the representativeness of the sample invited with respect to the target population by asking ‘Was the sample invited to participate in the study a true or close representation of the target population?’ The signalling question for this item asked about the method for recruitment of participants and, based on the response, the instructions guided the user to reach the corresponding RoB judgement (eg, low risk when the total or a randomly selected sample of the target population was invited; high risk for open calls for participation online or quota sampling; and unclear risk when the method to invite participants and the specific context of the sampling was not specified or when the target population was not defined; for more details, see the instructions in online supplemental appendix 2.

#### Item 2 selection bias: representativeness of the responders

The second item requires a judgement as to whether those who declined the invitation, in relation to those who participated in the study, would introduce bias in the prevalence estimate, ‘How would you rate the risk of non-response bias?’ The reasons for non-participation are instrumental in forming a judgement about RoB. However, these are rarely reported, if ever. We assumed that in our context the decision not to participate is associated, directly or indirectly with the mental health of the persons invited to the study. The signalling question for this item therefore inquires only about the participants providing data as a proportion of the number of people invited to participate. RoB judgement is based on the response.

#### Item 3 information bias: measurement of the condition

The third item assesses the likelihood of misclassification due to the methods used to measure the target condition, ‘How do you judge the risk of information bias?’ We provided guidance for judging this question for the MHCOVID project (online supplemental appendix 2); for instance, if the tool/method used to measure the condition was not applied properly across time points or across groups of participants, the risk of bias for this item was judged as high.

### Inter-rater agreement

Table 3 shows the results of the inter-rater agreement for each item of RoB-PrevMH, including both weighted and unweighted kappa for the 83 included studies. For item 1, the inter-rater agreement was substantial (weighted kappa 0.63, 95% CI 0.54 to 0.73) and overall agreement 83%. For item 2, the agreement was substantial (weighted kappa 0.71, 95% CI 0.67 to 0.85) and overall agreement 90%. For item 3, the weighted kappa was 0.32 (95% CI −0.04 to 0.63; overall agreement 93%), classifying inter-rater agreement as fair.

**Table 3:** Results of inter-rater agreement testing

Item	Unweighted kappa (95% CI)	% agreement	Weighted kappa (95% CI)	% agreement
1. Representativeness of the sampling frame	0.60 (0.48 to 0.68)	74.7	0.63 (0.54 to 0.73)	83.1
2. Representativeness of the responders	0.69 (0.59 to 0.70)	81.9	0.71 (0.67 to 0.85)	90.3
3. Measurement of the condition	0.28 (0.10 to 0.73)	89.2	0.32 (−0.04 to 0.63)	93.4

There was a total of 45 disagreements out of 249 paired assessments among 83 studies. Most of the disagreements (n=35) were between ‘unclear’ and either ‘high’ or ‘low’. Ten disagreements were between ‘high’ versus ‘low’ assessments.

## Discussion

### Summary of findings

We developed RoB-PrevMH, a concise RoB tool for prevalence studies in mental health that was designed with the intention to be adaptable to different systematic reviews and consisting of three items: representativeness of the sample, non-response bias and information bias. Our tool showed fair to substantial inter-rater reliability when applied to studies included in two systematic reviews of prevalence studies. All three items from RoB-PrevMH have been considered or included in existing tools.^14 18 21^ RoB-PrevMH does not contain any item on reporting and does not require an assessment of the overall RoB in a study. For each item, three assessments of RoB are possible (high, unclear and low)

#### Strengths and limitations

The strengths of RoB-PrevMH include the fact that it was created after a comprehensive review of items identified in previous tools as well as a consensus between researchers. Second, the feedback we received from the MH-COVID Crowd who used the tool suggests that the tool is concise and easy to use. Third, it focuses on RoB only and avoids questions that assess reporting. Fourth, the tool was tested by three pairs of extractors in two sets of studies with different aims. The inter-rater reliability was rated from fair to substantial. Finally, the tool has the potential to be tailored to other research questions.

Our tool also has limitations. First, the team of methodologists and investigators involved in development and testing was small. The tool would have benefited by a wider consultation strategy that involved more mental health experts and investigators who have designed and undertaken prevalence studies, as well as more methodologists. Second, the brevity of the tool could also be considered a limitation. For example, the MHCOVID project only includes studies that used validated tools for measuring mental health outcomes, so we did not include specific items for recall bias and observer bias, which might be important for other questions. Third, even if we assume that RoB-PrevMH would likely be quicker to complete than other tools, we did not formally assess the time required for completion in comparison with other tools. Fourth, the need to tailor the tool for each project and create training material for the people who will apply it might require more time than other tools at the start of a project. Moreover, the inter-rater reliability varied between the three items, with kappa values ranging from 0.32 to 0.71.

An important part of the evaluation of any RoB tool is the assessment of its validity. This is often done indirectly, by contrasting findings from studies judged at low versus high RoB in each domain. For example, randomised trials at high RoB from poor allocation concealment show, on average, larger effects than studies with low RoB.^26^ Prevalence studies are characterised by large heterogeneity, and it is expected that some of this heterogeneity might be associated with differences in RoB.^27^ However, RoB-PrevMH was not found to be associated with different study findings in a meta-analysis of the changes of symptoms of depression, anxiety and psychological distress during the pandemic, possibly because other design and population-related factors played a more important role in heterogeneity.^4^ A large-scale evaluation of the validity of RoB-PrevMH is needed to understand which design and analysis features impact most on the estimation of prevalence.

When we compare our tool’s performance with the available instruments, only the tool proposed by Hoy *et al* tested the inter-rater agreement and calculated the kappa value with a considerable number of studies on the prevalence of low back and neck pain.^14^ Even though representativeness of the target population might be difficult to judge objectively, the inter-rater agreement for this item was substantial while in the 54 studies assessed by Hoy *et al* the inter-rater agreement achieved was higher.^14^ For the second item on non-response, inter-rater agreement was substantial, but lower than similar items in the Hoy tool.^14^ The third item on misclassification had the lowest kappa statistic but the highest agreement between raters. In classification tables with great imbalance in the marginal probabilities and a high underlying correct classification rate kappa can be paradoxically low, as was the case of kappa for information bias.^28 29^ We did not make an overall RoB assessment for each study, which the Hoy tool does^14^ because of the problems with this approach.^24^

### Application of RoB-PrevMH in future projects

The design of prevalence studies differs substantially depending on the question they intend to answer; as a result, having a universal tool for all types of prevalence studies, like we have for RCTs and some observational studies,^23 30^ might not be realistic; instead, we need tools that can be tailored to specific research questions.^31^

Future projects applying RoB-PrevMH might need to improve the questions, and provide a more complete list of signalling questions and considerations to choose from, depending of the context and the nature of the measured prevalence. RoB-PrevMH was conceptualised and developed for the MHCOVID project,^4^ which required the use of a validated assessment tool. Additional questions about information bias might be needed for projects in which there are no validated diagnostic tools for a condition (eg, cognitive deficits in post-COVID-19) or the project does not impose inclusion criteria. Another example comes from the MHCOVID project itself. In this project we decided to rate RoB for the second and third item at every follow-up time point instead of following the original instructions to give one global rating for each study. Other projects might consider the idea of not having an arbitrary threshold for the proportion of respondents and instead extract the reported proportion and analyse the data by conducting prespecified subgroup analyses, based on this continuum of response rate with meta-regression. Moreover, our chosen arbitrary threshold for response rate might be inappropriate for other studies, as we included studies on the general population, during a pandemic and mostly done online; in other settings a ‘good’ response rate might be higher than 70%.

Evaluating the risk of information bias in prevalence studies of mental health problems requires special attention. The most reliable way to measure the presence of a condition is a diagnostic interview with a trained mental health professional; yet most studies use self-administered screening tools. These are questionnaires aiming to measure symptoms of the condition and the resulting score is used to infer about the presence or not of the condition. This, however, has been shown to overestimate the true prevalence.^32^ Consequently, care is needed in the interpretation of the prevalence estimated from such studies: the meta-analysis summary result cannot be interpreted as true prevalence of the condition, but rather as the prevalence of symptoms scores above the studied threshold.

Training for the tool should be tailored to a specific project and include relevant examples. For instance, for the MHCOVID project, we developed an educational video and provided online training for the volunteers of the project who extracted data from included studies and conducted RoB assessment (https://mhcovid.ispm.unibe.ch/crowd.html).

Assessment of RoB in prevalence studies applies to any condition. The tools that have been published were mostly developed for specific situations, ranging from low back pain to exposure to occupational risk factors. The methods that we used to develop RoB-PrevMH follow recommended methods for the development of guidelines^33^ and should be used to further develop an RoB tool that can be applied to any systematic review question that aims to summarise the prevalence of a condition or risk factor. The MHCOVID project has provided the basis for building a network or experts with experience of RoB assessment^23 30^ and critical appraisal of prevalence studies^9 16^ to develop a generic framework for tools to assess RoB in prevalence studies.^34^

## Conclusion

RoB-PrevMH is a brief and adaptable tool for assessing RoB in studies on PrevMH disorders. Initial results for inter-rater agreement were fair to substantial. The validity, reliability and applicability of RoB-PrevMH should be further assessed in future projects.

## References

[1] Leucht S , Cipriani A , Furukawa TA , et al . A living meta-ecological study of the consequences of the COVID-19 pandemic on mental health. Eur Arch Psychiatry Clin Neurosci 2021;271:219–21. 10.1007/s00406-021-01242-2 33675417 PMC7936586

[2] Liu Y-C , Kuo R-L , Shih S-R . COVID-19: the first documented Coronavirus pandemic in history. Biomed J 2020;43:328–33. 10.1016/j.bj.2020.04.007 32387617 PMC7199674

[3] Ipekci AM , Buitrago-Garcia D , Meili KW , et al . Outbreaks of publications about emerging infectious diseases: the case of SARS-Cov-2 and Zika virus. BMC Med Res Methodol 2021;21:50. 10.1186/s12874-021-01244-7 33706715 PMC7948668

[4] Salanti G , Peter N , Tonia T , et al . The impact of the COVID-19 pandemic and associated control measures on the mental health of the general population: a systematic review and dose-response meta-analysis. Ann Intern Med 2022;175:1560–71. 10.7326/M22-1507 36252247 PMC9579966

[5] Egger E , Higgins JPT , Davey Smith G . Systematic reviews in health research: meta-analysis in context, 3RD edition. In: Systematic Reviews in Health Research. Wiley, 2022. Available: Wileycom

[6] Viswanathan M , Berkman ND , Dryden DM , et al . Assessing risk of Bias and confounding in observational studies of interventions or exposures: further development of the RTI Item bank. Rockville (MD): Agency for Healthcare Research and Quality (US), 2013. 24006553

[7] Borges Migliavaca C , Stein C , Colpani V , et al . On behalf of the prevalence estimates reviews – systematic review methodology G: how are systematic reviews of prevalence conducted? A methodological study. BMC Med Res Methodol 2020;20:96. 10.1186/s12874-020-00975-3 32336279 PMC7184711

[8] Hoffmann F , Eggers D , Pieper D , et al . An observational study found large methodological heterogeneity in systematic reviews addressing prevalence and cumulative incidence. J Clin Epidemiol 2020;119:92–9. 10.1016/j.jclinepi.2019.12.003 31809847

[9] Borges Migliavaca C , Stein C , Colpani V , et al . Quality assessment of prevalence studies: a systematic review. J Clin Epidemiol 2020;127:59–68. 10.1016/j.jclinepi.2020.06.039 32679313

[10] Salanti G , Cipriani A , Furukawa TA , et al . An efficient way to assess the effect of COVID-19 on mental health in the general population. Lancet Psychiatry 2021;8:e14–5. 10.1016/S2215-0366(21)00067-5 33740412 PMC8824356

[11] Buitrago-Garcia D . Meta-análisis de prevalencia: revisión sistemática de los métodos utilizados, propuesta de una herramienta para evaluar la calidad y evaluación de los diferentes métodos estadísticos utilizados para meta analizar prevalencias. Bogotá, Colombia: Universidad Nacional de Colombia, 2018. Available: https://repositorio.unal.edu.co/handle/unal/63768

[12] Mesa-Vieira C , Haas AD , Buitrago-Garcia D , et al . Mental health of migrants with pre-migration exposure to armed conflict: a systematic review and meta-analysis. Lancet Public Health 2022;7:e469–81. 10.1016/S2468-2667(22)00061-5 35487232

[13] Shamliyan TA , Kane RL , Ansari MT , et al . Development quality criteria to evaluate nontherapeutic studies of incidence, prevalence, or risk factors of chronic diseases: pilot study of new Checklists. J Clin Epidemiol 2011;64:637–57. 10.1016/j.jclinepi.2010.08.006 21071174

[14] Hoy D , Brooks P , Woolf A , et al . Assessing risk of bias in prevalence studies: modification of an existing tool and evidence of Interrater agreement. J Clin Epidemiol 2012;65:934–9. 10.1016/j.jclinepi.2011.11.014 22742910

[15] Giannakopoulos NN , Rammelsberg P , Eberhard L , et al . A new instrument for assessing the quality of studies on prevalence. Clin Oral Investig 2012;16:781–8. 10.1007/s00784-011-0557-4 21594656

[16] Munn Z , Moola S , Riitano D , et al . The development of a critical appraisal tool for use in systematic reviews addressing questions of prevalence. Int J Health Policy Manag 2014;3:123–8. 10.15171/ijhpm.2014.71 25197676 PMC4154549

[17] Leboeuf-Yde C , Lauritsen JM . The prevalence of low back pain in the literature. A structured review of 26 Nordic studies from 1954 to 1993. Spine 1995;20:2112–8. 10.1097/00007632-199510000-00009 8588168

[18] Loney PL , Chambers LW , Bennett KJ , et al . Critical appraisal of the health research literature: prevalence or incidence of a health problem. Chronic Dis Can 1998;19:170–6. 10029513

[19] Boyle MH . Guidelines for evaluating prevalence studies. Evid Based Ment Health 1998;1:37–9. 10.1136/ebmh.1.2.37

[20] Silva LC , Ordúñez P , Paz Rodríguez M , et al . A tool for assessing the usefulness of prevalence studies done for surveillance purposes: the example of hypertension. Rev Panam Salud Publica 2001;10:152–60. 10.1590/s1020-49892001000900002 11702370

[21] Joanna Briggs I . The Joanna Briggs institute critical appraisal tools for use in JBI systematic reviews checklist for prevalence studies. Joanna Briggs Institute, 2017.

[22] Pega F , Norris SL , Backes C , et al . Rob-SPEO: A tool for assessing risk of bias in studies estimating the prevalence of exposure to occupational risk factors from the WHO/ILO joint estimates of the work-related burden of disease and injury. Environ Int 2020;135:105039. 10.1016/j.envint.2019.105039 31864023 PMC7479507

[23] Higgins JPT , Altman DG , Gøtzsche PC , et al . The Cochrane collaboration's tool for assessing risk of bias in randomised trials. BMJ 2011;343:d5928. 10.1136/bmj.d5928 22008217 PMC3196245

[24] Jüni P , Witschi A , Bloch R , et al . The hazards of scoring the quality of clinical trials for meta-analysis. JAMA 1999;282:1054–60. 10.1001/jama.282.11.1054 10493204

[25] Stroup DF , Berlin JA , Morton SC , et al . Meta-analysis of observational studies in epidemiology: a proposal for reporting meta-analysis of observational studies in epidemiology (MOOSE) group. JAMA 2000;283:2008–12. 10.1001/jama.283.15.2008 10789670

[26] Wood L , Egger M , Gluud LL , et al . Empirical evidence of bias in treatment effect estimates in controlled trials with different interventions and outcomes: meta-epidemiological study. BMJ 2008;336:601–5. 10.1136/bmj.39465.451748.AD 18316340 PMC2267990

[27] Migliavaca CB , Stein C , Colpani V , et al . Meta-analysis of prevalence: I^2^ statistic and how to deal with heterogeneity. Res Synth Methods 2022;13:363–7. 10.1002/jrsm.1547 35088937

[28] Cicchetti DV , Feinstein AR . High agreement but low Kappa: II. Resolving the paradoxes. J Clin Epidemiol 1990;43:551–8. 10.1016/0895-4356(90)90159-m 2189948

[29] Feinstein AR , Cicchetti DV . High agreement but low Kappa: I. The problems of two paradoxes. J Clin Epidemiol 1990;43:543–9. 10.1016/0895-4356(90)90158-l 2348207

[30] Sterne JA , Hernán MA , Reeves BC , et al . ROBINS-I: a tool for assessing risk of bias in non-randomised studies of interventions. BMJ 2016:i4919. 10.1136/bmj.i4919 27733354 PMC5062054

[31] Buitrago-Garcia D , Salanti G , Low N . Studies of prevalence: how a basic epidemiology concept has gained recognition in the COVID-19 pandemic. BMJ Open 2022;12:e061497. 10.1136/bmjopen-2022-061497 PMC9620521 36302576

[32] Thombs BD , Kwakkenbos L , Levis AW , et al . Addressing overestimation of the prevalence of depression based on self-report screening questionnaires. CMAJ 2018;190:E44–9. 10.1503/cmaj.170691 29335262 PMC5770251

[33] Moher D , Schulz KF , Simera I , et al . Guidance for developers of health research reporting guidelines. PLoS Med 2010;7:e1000217. 10.1371/journal.pmed.1000217 20169112 PMC2821895

[34] Buitrago-Garcia D . Development of a risk of bias tool for prevalence studies. 2023. Available: https://osf.io/b4qt9

